# Exploring the membrane topology of prohormone convertase 1 in AtT20 Cells: *in situ* analysis by immunofluorescence microscopy

**DOI:** 10.12688/f1000research.1-9.v1

**Published:** 2012-08-08

**Authors:** Niamh X Cawley, Meera Sridhar, Hong Hong, Peng Loh

**Affiliations:** 1 Section on Cellular Neurobiology, Program in Developmental Neuroscience, Eunice Shriver Kennedy National Institute of Child Health and Human Development, National Institutes of Health, Bethesda, MD, 20892, USA; 2 National Institute of Child Health and Human Development, National Institutes of Health, Bethesda, MD, 20892, USA; 3 The University of California, San Francisco, CA, 94143, USA

## Abstract

Prohormone convertase 1 (PC1) was previously characterized as a partially transmembrane protein in purified chromaffin granules of bovine adrenal medulla1. This was challenged with experiments on transfected PC1 in COS1 cells, a non-endocrine cell line2. To address this issue, we undertook to analyze its extraction properties *in vitro* and its immunocytochemical localization *in situ* in AtT20 cells, an endocrine cell line that expresses PC1. Most of the 87 kDa form of PC1 was resistant to carbonate extraction suggesting that it had properties of a transmembrane protein. Under semi-permeabilized conditions whereby only the plasma membrane was permeabilized, the carboxy-terminus of PC1 was specifically immunostained whereas the amino-terminus was not. These results indicate that the amino-terminus of PC1 was within the lumen of the Golgi and granules, and some of the C-terminus was exposed to the cytosol. Thus, endogenous PC1 can assume a transmembrane orientation *in situ* in AtT20 cells.

## 1. Introduction

The proprotein convertases (PCs) belong to a family of endoproteinases that cleave proproteins specifically at basic residue cleavage sites ^3^. The first mammalian member of this group, furin, was identified by sequence homology ^4, 5^ to the yeast prohormone convertase, Kex2, which was the first eukaryotic enzyme to be described as a prohormone convertase ^6, 7^. Both Kex2 and furin are transmembrane proteins ^5, 7– 9^. Other mammalian enzymes, homologous to furin were subsequently cloned, of which PC1 (also described as PC3 or SPC3) and PC2 were found to be exclusively expressed in (neuro)endocrine tissue ^10– 14^, suggesting their function to be specific for the maturation of peptide hormones and neuropeptides. Both enzymes do not contain classical amino acid sequences that would predict them to have a transmembrane domain.

PC1 is expressed as a pre-pro-protein of ~92 kDa in mass. After removal of the signal peptide, the pro-protein undergoes autocatalytic conversion in the ER to an 87 kDa form ^15– 18^. This form of PC1 can subsequently be converted to a 64–66 kDa form ^19, 20^ which is the predominant form found in dense-core granules of the bovine pituitary ^21^. The conversion from 87 kDa to 64 kDa is the result of the removal of the carboxyl terminus in a late compartment of the secretory pathway ^22^. We and others have investigated the function of the C-terminus of PC1, since it does not appear to be involved in catalysis per se and is distinct from the P-domain of PC1 which is involved in the stability and pH and calcium dependence of PC1 activity ^23^. Initial studies revealed that the C-terminus of PC1 is involved in the efficient trafficking of PC1 to the regulated secretory pathway (RSP) ^24– 26^, giving rise to the identification of three alpha-helical amphipathic sequences important in this function. Recent studies by Dikeakos *et al.* ^27, 28^, have characterized by NMR the extreme C-terminal sequence and identified important residues within the sequence involved in binding to membrane patches which were important for sorting.

Previously, we showed by immuno-labeling and classical extraction studies, that in intact purified bovine chromaffin granules, PC1 behaved in part like a transmembrane protein ^1^. We identified, immunologically and functionally, the putative transmembrane (TM) sequence (aa617–638), and showed that when fused with the soluble extracellular domain of the IL2 receptor alpha-subunit (Tac), it could direct this protein to secretory granules of the RSP ^1^. Indeed, deletion studies identified this sequence as necessary for PC1 sorting to the RSP ^29^. We speculated about how PC1 might assume a TM orientation and the consequences of having a cytosolic domain. However, Stettler *et al.* provided evidence that transfected PC1 is not synthesized as a TM protein in COS1 cells ^30^. In our current study we address in a model endocrine cell line, AtT20 cells, which expresses PC1 endogenously, whether PC1 has properties consistent with that of a TM protein.

## 2. Materials and methods

### 2.1 Cell culture

AtT20 and COS7 cells were grown in high glucose DMEM containing 10% fetal bovine serum, 1X penicillin/streptomycin and 50 μg/ml normocin (Invitrogen, San Diego, CA) in an incubator maintained at 37°C and 5% CO _2_. For sodium carbonate extraction, AtT20 cells were rinsed 3 times with ice cold phosphate buffered saline (PBS), scraped and collected in PBS, sedimented by centrifugation (3,000 rpm for 3 min) and resuspended in a smaller volume of PBS. The cell suspension was dispensed into equal aliquots and stored at -30°C until used.

### 2.2 Sodium carbonate extraction

A frozen aliquot of AtT20 cells was thawed on ice. One hundred μl of the cell suspension was saved immediately and 100 μl were placed into two airfuge tubes for centrifugation at 24 psi for 10 min, i.e. >100,000 × g (Airfuge, Beckman, Fullerton, CA). To block the non-specific binding of proteins to the plastic, the airfuge tubes were previously incubated on ice with 10% BSA for 30 min, after which they were rinsed 3 times with PBS. After centrifugation of the samples, the supernatants were removed and saved, and the pellets were resuspended in an additional 100 μl PBS each. The samples were centrifuged again and the resulting supernatants were combined with their original supernatants. To collect and save the pellet from the PBS extraction, the pellet from one tube was resuspended with 100 μl PBS and saved. The empty tube was rinsed once with 100 μl PBS and this was combined with the resuspended pellet. The other pellet was resuspended by vigorous pipetting with 100 μl of 0.1 M sodium carbonate, pH 11.5, and allowed to incubate on ice for 30 min. The sample was then centrifuged at 24 psi for 10 min and the resulting supernatant saved. The tube was rinsed carefully with 100 μl of fresh carbonate solution and this was added to the supernatant. The pellet was collected in PBS in the same way as was done for the first pellet. Thirty μl 1 M Tris/Cl, pH 7.4, 90 μl 4X SDS sample buffer and 36 μl 10X sample reducing agent were added to each of the extracted samples (containing 200 μl). To the original 100 μl aliquot of the starting material, half these volumes were added to maintain equivalent dilutions. Ten μl of the starting material and 20 μl of each extracted samples were analyzed by Western blot after SDS-PAGE through a 4–12% NuPAGE gel using the Bis-Tris buffer system and electro-blotting to nitrocellulose. Three transmembrane proteins; transferrin receptor, synaptotagmin 1 and aquaporin 1, and 3 non-transmembrane proteins; chromogranin A (β-granin), p115 and Grasp65 were analyzed. The blots were also probed for PC1 (N-terminal specific, ABR Inc., Golden, CO) in order to determine its pattern of extraction and to compare it to the patterns of the known transmembrane and non-transmembrane proteins listed above. Visualization of the proteins was by detection of secondary antibodies labeled with fluorophores that emit in the infra-red region of the spectrum using the Odyssey Infrared Imaging System (Licor Biosciences).

### 2.3 Generation and immunopurification of PC1 C-Terminal antibodies

For the detection of the C-terminus of PC1, a new antibody was generated against the peptide DSEDSLYSDYVDVFYN, which is present within the C-terminus of PC1 (amino acid numbering D _714_-N _729_). A cysteine residue was incorporated at the amino terminal to facilitate the coupling of the peptide to keyhole limpet hemocyanin (KLH). The synthesis of the peptide, coupling to KLH and generation of immune sera was performed under contract by Covance Research (Princeton, NJ). The antibodies were immunopurified as follows. Three mg of peptide (without KLH), dissolved in DMSO, were coupled to Affi-Gel 15 beads (Biorad, Hercules, CA) according to the manufacturer’s protocol. The beads were loaded into a column (~1.5 ml bed volume) and prepared for affinity chromatography. Five ml of the PC1 C-terminal antiserum (#5450) were mixed with 5 ml PBS containing 0.1% Tween 20 (PBST) and added to the column. The flow through sample was re-applied 3 times after which the column was washed with 30 ml PBST. The bound antibodies were eluted with 0.9 ml aliquots of 0.1 M Glycine, pH 2.9 into eppendorf tubes containing 100 μl 1 M Tris/Cl buffer, pH 7.5. Analysis by SDS-PAGE under reducing conditions and Coomassie Blue staining of the eluted fractions verified the presence of 50 kDa and 25 kDa immunoglobulin bands at an apparent purity estimated at >95% (data not shown). The purified IgGs were pooled and concentrated by centrifugation through 50 kDa molecular mass cutoff membranes (Pall Filtron, Northborough, MA). The buffer was also replaced with PBS/0.1% sodium azide by diafiltration through the same membranes. The resultant sample of immuno-purified IgGs (165 μg/ml) was stored at 4°C. These IgGs were used for the immunoprecipitation (IP) and immunocytochemistry (ICC) experiments.

### 2.4 Characterization of the purified IgGs by immunoprecipitation of PC1 from AtT20 cells

To demonstrate the specificity of the new C-terminal PC1 purified IgGs, an immunoprecipitation was performed using radio-labeled proteins from AtT20 cells. The cells were labeled for 24 h with a mixture of [ ^35^S]-Met/[ ^35^S]-Cys (100 μCi/ml). Following this, the cells were rinsed 3 times with ice cold PBS and then harvested in 50 mM Tris-Cl (pH 7.5), 150 mM NaCl, 2 mM EDTA (TNE buffer) containing 0.1% Triton X-100 and freshly prepared phenyl-methylsulfonyl fluoride (PMSF, 1 mM). The homogenate was centrifuged at 13,000 rpm for 10 min to sediment insoluble cellular material and a second extraction was performed on this pellet. The supernatants from both extractions were combined and incubated at 60°C for 20 min after addition of SDS to a final concentration of 0.1%. Following centrifugation of the sample (13,000 rpm, 10 min), Triton X-100 was added to the supernatant to a final concentration of 1%. The sample was pre-cleared by addition of protein A-sepharose beads (50 μl of a 50% slurry, 30 min, 4°C). After centrifugation to remove the beads, the supernatant was incubated with 1 μg of PC1 C-terminal immuno-purified IgGs or 1 μg of PC1 N-terminal immuno-purified IgGs for 18 h at 4°C. Antibody:antigen complexes were precipitated with 30 μl of protein-A sepharose beads and were washed extensively. The beads were resuspended in 1X tris-glycine SDS sample buffer containing β-mercaptoethanol, boiled for 10 min to elute the proteins and then analyzed by autoradiography after SDS-PAGE and electroblotting onto PVDF membrane. An additional gel was run later for the analysis of the IP supernatant. In that case, a 4–12% NuPAGE gel was used and the proteins transferred to nitrocellulose for autoradiography.

### 2.5 PC1 topology by immunocytochemistry

It was demonstrated previously in purified bovine adrenal chromaffin granules that some PC1 could adopt a transmembrane orientation ^1^. This suggested that some of the C-terminus of PC1 is localized on the cytosolic side of secretory granules in the cell. In order to investigate this, *in situ*, we employed a procedure that has previously been characterized ^31^ and one which we had been investigating independently. This procedure utilizes the observation that fixation of cells with *para*-formaldehyde (PFA) in PBS selectively permeabilizes the plasma membrane and allows access by immunoglobulins to the cytosolic space. Thus, cytosolic epitopes would be accessible for immunofluorescence microscopy in PFA/PBS fixed cells. On the other hand, proteins within the lumen of organelles, such as those found within the secretory pathway would not be accessible ^31^. Using this procedure it is therefore possible to demonstrate the topology of a transmembrane protein within cells *in situ* if domain specific antibodies were available.

AtT20 cells were grown in two-chambered glass slides, rinsed 3 times with room temperature (RT) PBS and then fixed in 2% PFA/PBS for 30 min at RT. One set of chambers was permeabilized by 0.25% Triton X-100/PBS for 5 min at RT while the other set received only PBS. After blocking with 1% bovine serum albumen (BSA)/PBS for 2 h at RT, the cells were then incubated for 16–20 h at 4°C with primary antiserum diluted as indicated in 1% BSA/PBS. Primary antibodies used were as follows; mouse anti-transferrin receptor (1:1,000, cytoplasmic epitope) (Invitrogen, Carlsbad, CA), mouse anti-p115 (1:1,000) (BD Biosciences, San Jose CA), rabbit anti-GRASP65 (1:2,000) (Proteus Biosciences, Ramona, CA), mouse anti-ACTH (1:1,000) (Abcam, Cambridge, MA), rabbit anti-PC1 (10 μg/ml, N-terminal specific) (Affinity Bioreagents Inc., Golden, CO) and rabbit anti-chromogranin A (1:10,000) ^32^. The rabbit anti-PC1 (C-terminal specific) immuno-purified antibody, which was generated in our laboratory (see above), was used at a concentration of 1.6 μg/ml. This antibody was also used in combination with the mouse anti-p115 in a double labeling experiment. To demonstrate specificity, the C-terminal specific PC1 purified antibodies were pre-absorbed with the immunogenic peptide (1 μg/ml) and also used. After extensive washing with PBS, primary antibodies were detected with Alexa dye-conjugated secondary antibodies; goat anti-rabbit-568 (1:1,000) or goat anti-mouse-488 (1:1,000) from Molecular Probes (Invitrogen, Carlsbad, CA). All pictures were captured on an LSM 510 inverted scanning confocal microscope in the NICHD Microscopy and Imaging Core facility. For each antigen, power settings were optimized for the Triton X-100 (TX-100) treated cells until a clear, strong picture was obtained. These settings were then used to detect the same antigen in the non TX-100 treated cells, so that a direct comparison could be made between the staining intensities of the same antigen under the two conditions.

Prohormone convertase 1 cDNA ^29^, encoded in the mammalian expression vector, pcDNA3.1, was transfected into COS7 cells using Lipofectamine 2000 according to the manufacturer (Invitrogen). Forty-eight h after transfection, the cells were processed for ICC under TX-100 treated and untreated conditions and images captured as described above.

## 3. Results

### 3.1 Sodium carbonate extraction

Three transmembrane proteins; transferrin receptor, aquaporin 1 and synaptotagmin 1, were studied as a set of control proteins for the classical procedure of alkaline sodium carbonate extraction. All 3 of these proteins were predominantly recovered in the sodium carbonate pellet indicative of their resistance to extraction by this procedure ( Figure 1). Three non-transmembrane proteins were also studied as another set of control proteins for this procedure. These proteins, β-actin, chromogranin A (or β-granin when processed) and p115, a protein peripherally associated with the cytoplasmic side of the Golgi ^33^, were recovered in the PBS supernatant ( Figure 1). Residual levels of these proteins that were recovered in the PBS pellet were subsequently extracted in the sodium carbonate supernatant, demonstrating, as non-transmembrane proteins, their susceptibility to extraction by this procedure. These 6 proteins were established as positive and negative controls for the carbonate extraction procedure of AtT20 cells and all volumes were maintained equivalently so that a direct relative quantification of the proteins recovered at each step could be obtained when compared to the starting material. The distribution of PC1 was also analyzed using an N-terminal specific PC1 antibody. It was found that the 64 kDa form of PC1 was predominantly recovered in the PBS supernatant whereas the 87 kDa form was recovered in the PBS pellet. The 87 kDa form was subsequently recovered predominantly in the sodium carbonate pellet along with a small amount of the 64 kDa form ( Figure 1).

**Figure 1.**
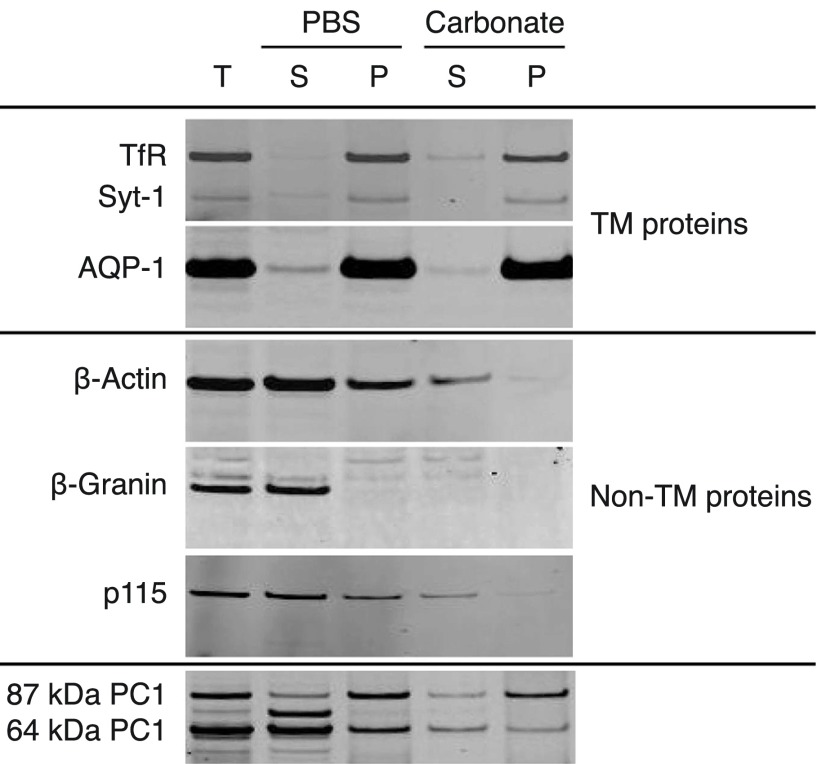
Carbonate extraction of AtT20 cells. AtT20 cells were subjected to extraction by PBS followed by 0.1 M sodium carbonate, pH 11.5. Equivalent volumes from each step were analyzed by Western blot. Three TM proteins (transferrin receptor (TfR), synaptotagmin 1 (Syt-1) and aquaporin 1 (AQP-1) and 3 non-TM proteins (β-actin, β-granin and p115) were analyzed as controls. The 3 TM proteins were recovered in the sodium carbonate pellet while the 3 non-TM proteins were predominantly recovered in the PBS supernantant. The distribution of PC1 was also analyzed. The 64 kDa form was predominantly recovered in the PBS supernatant while the 87 kDa form (and a small amount of the 64 kDa form) was predominantly recovered in the carbonate pellet. This suggested that the 87 kDa form and some of the 64 kDa form of PC1 have properties consistent with a TM protein. T, total; S, supernatant; P, pellet.

### 3.2 Immunoprecipitation of PC1 from AtT20 cells

Under steady state conditions, 2 forms of PC1 are found in AtT20 cells, an 87 kDa form and a 64 kDa form, both of which have an identical N-terminus. Immunoprecipitation by the N-terminal specific IgGs resulted in the capture of both these forms ( Figure 2, N-term). When the C-terminal specific IgGs were used, one major band was captured consistent with being the 87 kDa PC1 form as it co-migrated with the 87 kDa form captured by the N-terminal specific IgGs ( Figure 2, C-term). A faint band, with an apparent molecular mass of ~20 kDa based on the molecular mass standards (SeeBlue Plus 2, Invitrogen) was also seen. This band was considered likely to be the processed carboxyl terminus of PC1 since it was only present in the C-terminal specific IP lane and it has the same molecular mass as a previously expressed form of the C-terminal domain of mouse PC1 ^34^. Western blot analysis of a subsequent IP of unlabeled AtT20 cells (both carried out with the PC1 C-terminal IgGs), failed to show such a PC1 immunoreactive protein (data not shown), indicating that it’s levels were too low for Western blot detection (compared to the radiolabeled band) or was possibly a protein that co-immunoprecipitated with the 87 kDa form of PC1.

**Figure 2.**
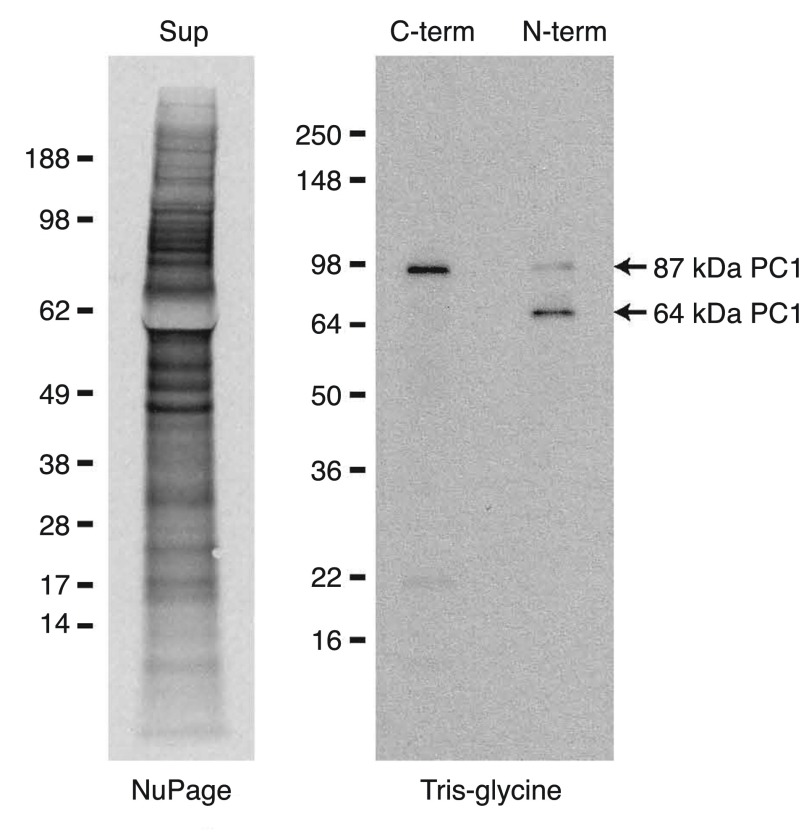
Immunoprecipitation of PC1 from AtT20 cells. To demonstrate the specificity of the immuno-purified PC1 C-terminal specific IgGs, an immunoprecipitation (IP) was performed on radiolabeled AtT20 cells. From a multitude of labeled proteins (Sup lane), one major band at 87 kDa was immunoprecipitated with these IgGs (C-term lane). As a control, immunoprecipitation with N-terminal specific PC1 IgGs yielded the two expected bands of 87 kDa and 64 kDa PC1 (N-term lane). A faint band at ~20 kDa was deemed non-specific as it was not immuno-reactive with the C-terminal specific IgGs in a Western blot of a subsequent IP of unlabeled AtT20 cell lysate (data not shown).

### 3.3 PC1 topology by immunocytochemistry

To demonstrate that PFA fixation selectively permeabilizes the plasma membrane, we performed ICC on TX-100 treated and non-treated AtT20 cells and analyzed the staining pattern of 6 endogenous proteins; 3 with epitopes localized in the cytosol and 3 proteins localized within the lumen of organelles belonging to the regulated secretory pathway (RSP) which includes the Golgi and secretory granules. For all 3 lumenal proteins, CgA, ACTH and the N-terminus of PC1, strong staining of the Golgi and tips of the processes were observed only in the TX-100 treated cells consistent with their presence in the RSP ( Figure 3A-C). In the absence of TX-100, no staining of these proteins could be detected ( Figure 3D-F), demonstrating the requirement for the detergent to expose these proteins to the antibodies by permeabilizing the membranes of the organelles. For the 3 proteins with cytosolic epitopes, p115, Grasp 65 and transferrin receptor, strong immuno-staining was observed whether TX-100 was used or not ( Figure 4). Thus PFA fixation allows accessibility of the IgGs to the cytosol, where they can bind their antigens, but not to the lumen of the organelles of the RSP.

**Figure 3.**
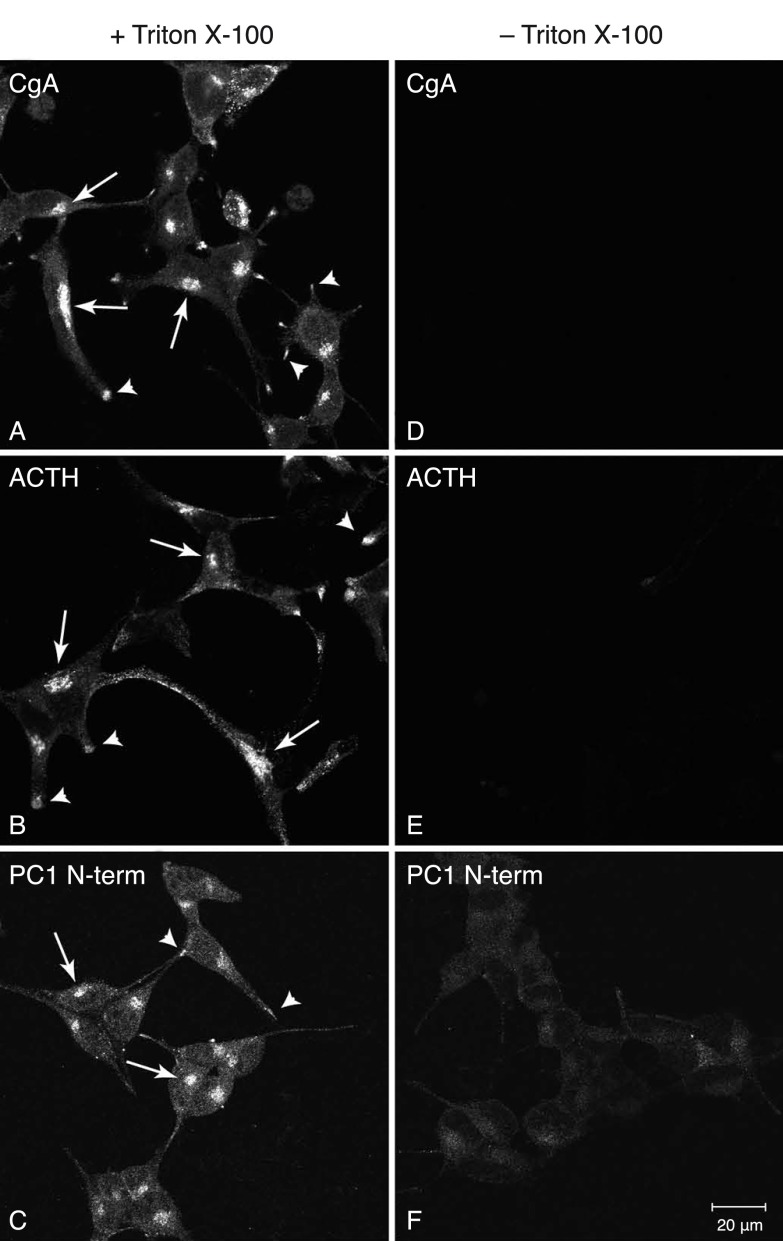
Immunocytochemical analysis of RSP luminal proteins in AtT20 cells. AtT20 cells were chemically fixed with 2% PFA/PBS and then treated with and without the detergent, Triton X-100. Three luminal proteins belonging to the RSP (Chromogranin A, ACTH and the N-terminus of PC1) were stained by indirect immunofluorescence. For all three proteins, the Golgi (arrows) and the tips of the processes (arrow heads) were specifically stained when Triton X-100 was used ( **A**– **C**). No staining was seen when Triton X-100 was not used ( **D**– **F**). This staining pattern is consistent with the presence of these proteins within the organelles of the RSP and demonstrates that PFA fixation does not cause an access of the antibodies to these compartments. Bar 20 μm

**Figure 4.**
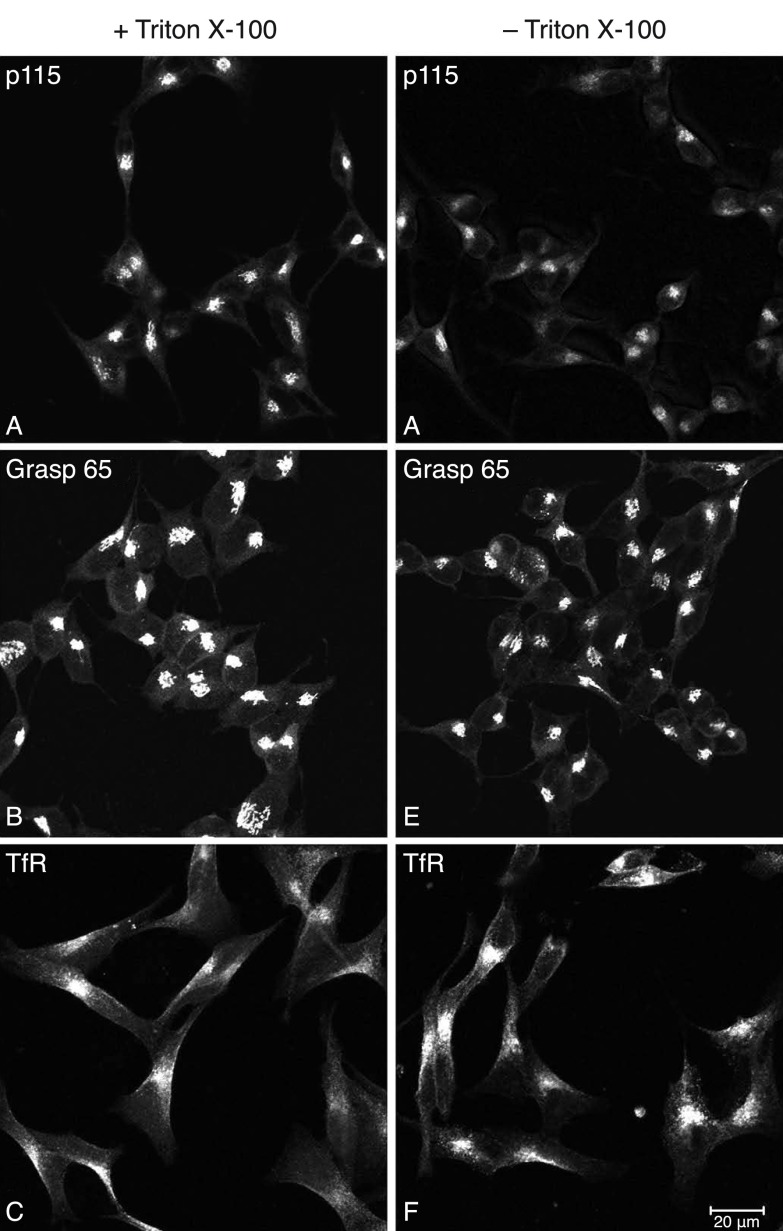
Immunocytochemical analysis of cytosolic proteins in AtT20 cells. AtT20 cells were chemically fixed with 2% PFA/PBS and then treated with and without the detergent, Triton X-100. Three cytosolic proteins (p115, Grasp65 and N-terminus of the transferrin receptor) were stained by indirect immunofluorescence. For p115 ( **A**, **D**) and Grasp65 ( **B**, **E**), staining of the Golgi area was observed and for the transferrin receptor ( **C**, **F**), staining of the plasma membrane/endosomes were observed, whether the cells were treated with Triton X-100 or not. This demonstrated that the antibodies had access to the cytosol even with only PFA fixation. Bar 20 μm

Using this procedure with the C-terminal specific immuno-purified PC1 antibodies, a pattern of staining for PC1 was observed in the TX-100 treated cells that was similar to that of CgA, ACTH and the N-terminal specific PC1 antibodies, i.e. strong staining of the Golgi and a punctate pattern in the processes ( Figure 5, top panel, PC1). The staining pattern exhibited by these purified antibodies is consistent with the localization of PC1 in the Golgi, as evidenced by its colocalization with p115 ( Figure 5, top panel, p115 and Merged) and secretory granules of the RSP. No staining could be seen when immuno-purified C-terminal PC1 IgGs were used that had been pre-absorbed by the antigenic peptide ( Figure 5, Absorption control). In the absence of TX-100, however, while staining with the N-terminal specific PC1 antibodies was negative ( Figure 3F); staining of the Golgi and processes was observed in the untreated cells with the C-terminal specific IgGs ( Figure 5, lower panel, PC1). This pattern of staining indicated that the C-terminus of PC1 is present in the cytosol and the N-terminus of PC1 is in the lumen of the Golgi and secretory granules, indicating that at least some PC1 is in a transmembrane orientation *in situ* and supports the results of the extraction experiments ( Figure 1).

**Figure 5.**
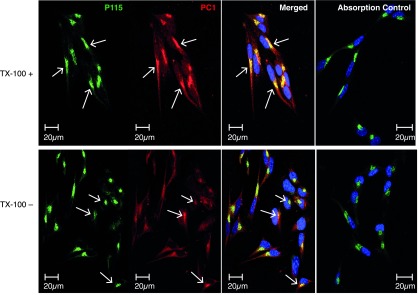
Immunocytochemical analysis of PC1 C-terminus in AtT20 cells. AtT20 cells were chemically fixed with 2% PFA/PBS and then treated with and without the detergent, Triton X-100. PC1 was stained with C-terminal specific immunopurifed IgGs by indirect immunofluorescence. In the Triton X-100 treated cells, the staining pattern of the C-terminus of PC1 was similar to that of the N-terminus of PC1 described in Figure 3, in that Golgi staining (arrows) was observed (top panel). In the Triton X-100 untreated cells, however, a reduced but similar staining pattern was observed to that of the Triton X-100 treated cells (lower panel). This demonstrates that some of the C-terminus of PC1 was localized in the cytosol. Bar 20 μm.

The topology of PC1 transfected into non-endocrine COS7 cells was assessed also by this procedure. After fixation by PFA, the C-terminus of PC1 was strongly stained by the C-terminus specific purified IgGs only after permeabilization with TX-100 ( Figure 6A). This result indicated that PC1 did not assume a TM orientation in COS7 cells consistent with the results of Stettler *et al.* in COS1 cells ^30^.

**Figure 6.**
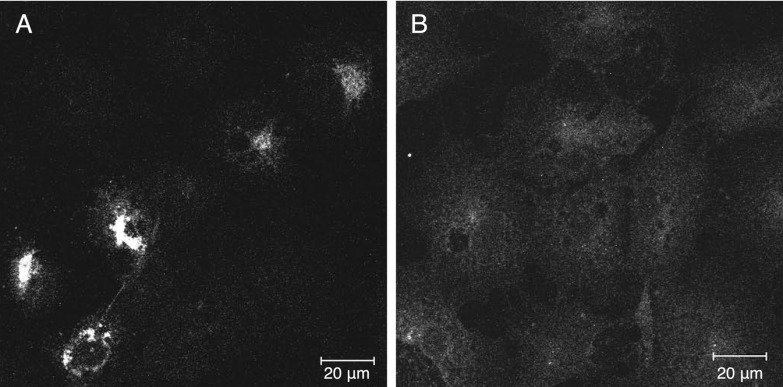
Immunocytochemical analysis of PC1 C-terminus in COS7 cells. COS7 cells, expressing transfected full length PC1, were chemically fixed with 2% PFA/PBS and then treated with and without the detergent, Triton X-100. PC1 was stained with C-terminal specific immunopurifed IgGs by indirect immunofluorescence. In the Triton X-100 treated cells, a strong staining pattern of the C-terminus of PC1 was observed in the transfected cells consistent with a distribution in the reticular network of the ER and in the Golgi. ( **A**) Only a low level of background staining was observed for the TX-100 untreated cells ( **B**). Bar 20 μM.

## 4. Discussion

Prohormone convertase 1 (PC1) is sorted to the regulated secretory pathway (RSP) of (neuro)endocrine cells where it functions to cleave prohormones and proneuropeptides into smaller peptides that ultimately function in important biological processes. How PC1 is sorted to the RSP has been actively studied and several proposals have been put forward. A commonality among these ideas is the belief that association of the C-terminal tail of PC1 with components of the trans-Golgi network (TGN) membrane, where sorting to the RSP is believed to be initiated, must occur, although binding via the prodomain has also been implicated ^35^. In light of the extraction and/or binding studies by Hill *et al.* ^21^, Arnaoutova *et al.* ^1^ and Jutras *et al.* ^25^, it is considered that such binding or membrane association is quite strong. Without evidence of amino acid sequences that predict a classical transmembrane (TM) domain similar to furin and Kex2, and with the previously identified membrane binding amphipathic α-helices within the C-terminus ^25^, it is reasonable to expect that binding would be to the luminal side of the TGN membrane in a non-TM manner and that such binding would be necessary for sorting to the RSP.

However, we have previously studied the membrane association properties of carboxypeptidase E (CPE) that contains an amphipathic α-helix at its C-terminus ^36, 37^ and demonstrated that it can assume a transmembrane topology in a lipid raft dependent manner. Indeed, live cell imaging demonstrated a role of its cytoplasmic tail in peptide hormone granule transport via interaction with dynactin and the microtubule dependent motor proteins, kinesin and cytoplasmic dynein ^38, 39^. Hence, based on these and other observations of the novel TM behavior of CPE and PC2 via their C-termini ^37, 40^, we speculated that lipid raft-association and TM orientation of PC1 might occur through a similar C-terminal domain that was identified in PC1. Thus, in our previous work we tested this idea principally in intact purified, bovine adrenal medulla chromaffin granules and showed the presence of the C-terminus of PC1 on the outside of these granules. In such a case the cytosolic C-terminus would be quite long (~114 aa). To explain how this might occur, we speculated that, since insertion through the membrane in the Golgi or post-Golgi compartment would be energetically unlikely, PC1 might be synthesized as a TM protein ^1^.

Our speculations, however, have been challenged by Stettler *et al.* who showed by various methods that transfected PC1 is not synthesized as a TM protein in COS1 cells ^30^. The results of Stettler *et al.* were important for two reasons. One, it provided information about the initial synthesis of PC1, albeit in a non-endocrine cell, and two; it questioned whether PC1 was a TM protein at all, because the data by Arnaoutova *et al.* which demonstrated it to be a TM protein in intact purified granules, was discounted by Stettler *et al.* simply as the result of contamination. While this is a valid point, we do not consider it to be probable because, had there been even a small amount of contamination, chromogranin A, the most abundant protein in chromaffin granules (47% of soluble granule content, 7% of total adrenal medulla content ^41^, would, like PC1, also have been biotinylated when the purified granules were used in the biotinylation experiment. The observation that PC1 was biotinylated and CgA was not provided very strong evidence that PC1 was there in a transmembrane orientation rather than by contamination from ruptured organelles.

Regardless of this explanation and to readdress the issue of PC1 topology, we undertook to analyze endogenously expressed PC1 in a model endocrine cell line where both forms are known to exist at steady state. Initial extractions by sodium carbonate, pH 11.5, a classical procedure for the characterization of TM proteins initially described by Fujiki *et al.* ^42^, suggested that the 87 kDa form (and some of the 64 kDa form) of PC1 had properties of a TM protein because its partitioning behavior was similar to three known TM proteins ( Figure 1). These results are consistent with the previously published data on PC1 from bovine chromaffin granules ^1^ and indicated to us that PC1 in AtT20 cells had similar properties to PC1 found in chromaffin cells from bovine adrenal medulla. While resistance to alkaline sodium carbonate extraction is not definitive proof of TM orientation, it is considered to be strong evidence for such a conclusion. To investigate this further we studied PC1 topology in AtT20 cells under steady state conditions *in situ* by immunofluorescence confocal microscopy. This simple but powerful procedure is based on the observation that fixing cells for immunocytochemistry (ICC) with *para*-formaldehyde (PFA) in PBS, permeabilizes the plasma membrane sufficiently to allow immunoglobulins (IgGs) into the cytosol ^31^. However, since PFA/PBS does not have the same effect on membranes of internal organelles, we can determine the topology of organellar proteins if domain specific IgGs are available.

To demonstrate the validity of this technique in AtT20 cells, we performed ICC on PFA fixed cells with and without detergent permeabilization and analyzed the staining of 3 known cytosolic proteins (Grasp65, p115 and the N-terminus of the transferrin receptor) and 3 known luminal proteins belonging to the regulated secretory pathway (RSP) (ACTH, CgA and the N-terminus of PC1). As expected, the RSP proteins were only stained when the cells were permeabilized with the detergent, Triton X-100 (TX-100) demonstrating the integrity of the membranes of the internal organelles ( Figure 3). The cytosolic proteins were stained whether TX-100 was used or not ( Figure 4) demonstrating that the antibodies had access to the cytosol even in the absence of detergent treatment. Staining of the C-terminus of PC1 with our immuno-purified IgGs gave a similar pattern of staining to that of the PC1 N-terminal specific IgGs when performed on TX-100 treated cells. In the absence of TX-100, however, reduced but specific staining with the C-terminal specific IgGs was also observed indicating that some of the C-terminus of PC1 was present in the cytosol ( Figure 5). Golgi staining of the C-terminus was observed (as demonstrated by its colocalization with p115) as well as punctate staining in the processes, indicative of granules, suggests that PC1 (or some of it) can be in a TM orientation in the Golgi and granules.

How this happens is currently unknown. However, assuming that the results observed by Stettler *et al.* in the COS1 cells are similar for the synthesis of endogenous PC1 in classical (neuro)endocrine tissue/cell lines, we are now directed to re-consider the possibility that an insertion event might be taking place after synthesis in the ER. Although the sequence identified as the TM domain (aa619–638) ^1, 29, 43^ does not have classical TM characteristics, it is conceivable that one or several factors that are still unknown may facilitate or stabilize PC1 in such an insertion/orientation, thus reducing the free energy necessary for such an event. An example of a "helper" protein exists for the diphtheria toxin where insertion into model membranes as a TM protein only occurs in the presence of molten globule-like proteins ^44^. In addition, while it is known that C-terminal tail-anchored proteins (TA proteins, e.g. cytochrome b5) are TM proteins, the mechanism by which membrane insertion of these cytosolic proteins occurs is still unknown as it is independent of the Sec61 translocon ^45^.

It is probable that not all of the PC1 becomes TM, in which case an equilibrium may exist between luminal/peripheral and TM partitioning and that this equilibrium may depend on levels of endogenous factors as indicated above. Indeed a more intense signal of the PC1 C-terminal signal in the granules was observed in the TX-100 treated cells ( Figure 5, top panel versus lower panel) indicating the presence of the C-terminus within the lumen of the granules presumably as a cleaved product. The concept of "helper" proteins is also supported by our observations that when we transfected PC1 into COS7 cells ( Figure 6) or PC12 cells (a model neuroendocrine cell line) (data not shown) and performed the ICC experiment we could only observe specific PC1 C-terminal staining when TX-100 was used indicating that in transfected cells the PC1 did not measurably adopt a TM orientation. This suggested to us that TM insertion is a saturable process that appears to require components that are limiting in (neuro)endocrine cells or not present in non-endocrine cells. Studies are underway to identify such components.
